# Supplementary figures and images for: The gammaherpesvirus 68 viral cyclin facilitates expression of LANA
Source: PLoS Pathog. 2021 Nov 15;17(11):e1010019. doi: 10.1371/journal.ppat.1010019 (PMC8629379; doi:10.1371/journal.ppat.1010019)

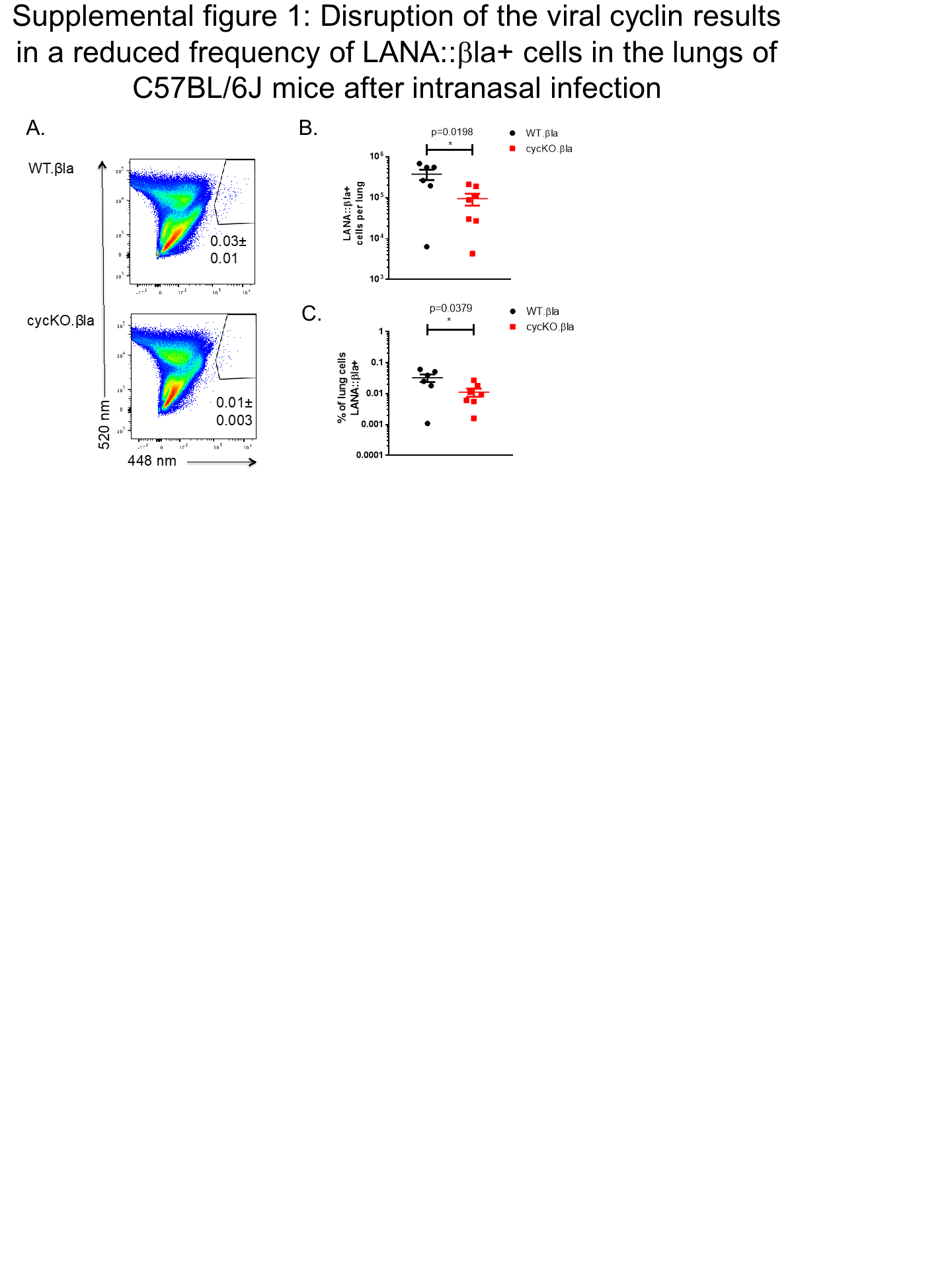

Supplement: S1 Fig — Mice were infected via I.N. inoculation with WT.βla or cycKO.βla viruses and lung were harvested at 8 dpi. (A) Representative pseudocolor plots identifying βla+ lung cells in the upper right polygon. Frequency of βla+ cells is indicated below the gate +/- SEM. (B) The percent of lung cells that are βla+ for each mouse is plotted with SEM shown after infection with WT.βla (black) or cycKO.βla (red). (C) The total number of βla+ cells per lung for each mouse is plotted with SEM shown after infection with WT.βla (black) or cycKO.βla (red). WT.βla n = 6 cycKO.βla n = 7. Two-tailed student t test was used for statistical analysis. (TIF) [file ppat.1010019.s001.tif]
